# Comparison of Surgically Induced Astigmatism Between Baerveldt Glaucoma Implant Surgery and Trabeculectomy: A Retrospective Cohort Study

**DOI:** 10.3390/jcm15041620

**Published:** 2026-02-20

**Authors:** Kengo Tanaka, Kentaro Iwasaki, Shogo Arimura, Marie Suzuki, Yoshihiro Takamura, Masaru Inatani

**Affiliations:** Department of Ophthalmology, Faculty of Medical Sciences, University of Fukui, Fukui 910-1193, Japan; kengot@u-fukui.ac.jp (K.T.); leosunshine33@gmail.com (S.A.); marie.ohgoshi.0408@gmail.com (M.S.); ytakamura@hotmail.com (Y.T.); inatani@u-fukui.ac.jp (M.I.)

**Keywords:** Baerveldt glaucoma implant, trabeculectomy, surgically induced astigmatism, keratometry, vector analysis, filtering bleb

## Abstract

**Objectives**: This study aimed to compare surgically induced corneal astigmatism (SIA) between Baerveldt glaucoma implant (BGI) surgery and trabeculectomy and to identify prognostic factors associated with SIA. **Methods**: This retrospective cohort study included 109 and 229 eyes that underwent BGI surgery and trabeculectomy, respectively, at Fukui University Hospital. Corneal astigmatism was assessed preoperatively, and at 6 months postoperatively, the mean SIA (M-SIA) and centroid SIA were calculated, and factors associated with M-SIA were determined. **Results**: BGI surgery was associated with greater SIA than trabeculectomy. Postoperative corneal astigmatism was −1.53 ± 1.01 D in the BGI group and −1.33 ± 0.92 D in the trabeculectomy group (*p* = 0.044). The M-SIA was significantly larger in the BGI group (1.41 ± 1.05 D vs. 0.94 ± 0.74 D, *p* < 0.01). Multivariate regression revealed that BGI surgery and lower preoperative corneal astigmatism were independently associated with greater M-SIA. Subgroup analysis revealed that older age and lower preoperative astigmatism were significant predictors of larger M-SIA in the BGI group, whereas only preoperative astigmatism was significant in the trabeculectomy group. **Conclusions**: BGI surgery was associated with greater and more variable SIA than trabeculectomy. Surgical type, baseline astigmatism, and age are important predictors of postoperative SIA.

## 1. Introduction

Surgically induced corneal astigmatism (SIA) is an important consideration in glaucoma surgery because postoperative changes in corneal shape can affect visual quality and ocular refractive status. Trabeculectomy is the most widely performed filtration surgery; however, it causes postoperative astigmatic changes, resulting in decreased visual acuity [1,2,3,4,5,6,7,8,9,10,11,12,13]. In contrast, glaucoma drainage device surgery using the Baerveldt glaucoma implant (BGI) has become increasingly common for refractory glaucoma [14]; however, corneal curvature can be influenced by the biomechanical impact of factors such as large implant plate, tube entry site, and postoperative tissue response. Despite these concerns, only few studies have directly compared SIA between BGI and trabeculectomy.

Moreover, the pattern and magnitude of SIA may vary depending on bleb location, surgical technique, and individual ocular biomechanics. Notably, several mechanisms underlying the development of SIA after trabeculectomy have been proposed, including scleral suturing [2,3,13], excessive cautery [1,2,3], pressure from the eyelid or filtering bleb [8], delayed wound healing due to mitomycin C [4], lower postoperative intraocular pressure (IOP) [6,8,10], and the surgical site [10]. Vector analysis, including the arithmetic mean SIA (M-SIA) and centroid SIA (C-SIA), comprehensively evaluates the magnitude and direction of astigmatic changes. SIA after trabeculectomy has been investigated; however, the difference in SIA between trabeculectomy and BGI surgery, or the clinical factors associated with greater postoperative astigmatic change, is unclear.

The purpose of this study was to compare SIA between BGI surgery and trabeculectomy using vector analysis and to identify clinical factors associated with greater postoperative astigmatic change.

## 2. Materials and Methods

### 2.1. Ethics Declarations

This retrospective clinical cohort study was approved by the Institutional Review Board of Fukui University Hospital. The study protocol adhered to the tenets of the Declaration of Helsinki, and the requirement for informed consent was waived owing to the retrospective nature of the investigation.

### 2.2. Patient Selection

This study included patients aged ≥ 20 years who had undergone BGI (BG101-350 or BG102-350; Johnson & Johnson Vision, Irvine, CA, USA) surgery or trabeculectomy between 1 April 2012 and 31 March 2023, at Fukui University Hospital. The inclusion criteria were as follows: patients in whom the endplate was placed in the superotemporal quadrant during BGI surgery and those in whom a scleral flap was created in the superior or superotemporal quadrant during trabeculectomy. The exclusion criteria were as follows: patients with no light perception, those who had previously undergone tube shunt surgery, those who underwent combined cataract surgery, those without a follow-up period of ≥6 months postoperatively, those who developed severe complications, such as bleb leak or hypotony at 6 months postoperatively, those requiring additional interventions within 6 months postoperatively, except for laser suture lysis (LSL), bleb needling, or anterior chamber washing, and those with a history of intraocular surgery within 6 months before or after the primary surgery. Eyes that wore hard contact lenses until immediately before surgery were also excluded. We analyzed only eyes with preoperative and 6-month postoperative keratometric measurements. When both eyes of the same patient met the inclusion criteria, only the first-operated eye was included in the analysis. In eyes that underwent both trabeculectomy and BGI surgery, only the initial procedure was included.

### 2.3. Surgical Procedures

#### 2.3.1. Baerveldt Glaucoma Implantation

All tube shunt procedures were performed using BG101-350 or BG102-350 end plates. The silicone tube was ligated using an 8-0 absorbable vicryl suture to prevent early postoperative hypotony. A fornix-based conjunctival flap was created after subconjunctival and sub-Tenon’s anesthesia with xylocaine. The implant plate was positioned in the superotemporal quadrant and secured to the sclera approximately 10 mm posterior to the corneal limbus, and placed under the rectus muscles. A scleral tunnel was created using a 23-gauge needle, and the tube was inserted into the anterior chamber or ciliary sulcus. In eyes with a history of vitrectomy, the tube was inserted into the vitreous cavity through the pars plana. For pars plana tube insertion, the tube with the Hoffmann elbow (BG102-350) or straight tube (BG101-350) was inserted into the vitreous space using a 20- or 24-gauge microvitreoretinal-lance. Sherwood slits were created in the tube using a 9-0 nylon needle to reduce the incidence of early postoperative IOP elevation. The tube was covered with a half-thickness rectangular self-scleral flap or a preserved scleral patch graft obtained from the eye bank. The scleral patch and conjunctival flap were sutured using 9-0 nylon and 8-0 absorbable vicryl sutures.

#### 2.3.2. Trabeculectomy

Trabeculectomy was performed using either a fornix- or limbus-based conjunctival approach. In the fornix-based technique, a conjunctival incision of approximately 5 mm was made along the limbus, whereas in the limbus-based technique, an 8 mm incision was made parallel to the limbus at a distance of 7–9 mm posterior to it. A 4 mm wide half-thickness scleral flap (triangle or square) was subsequently created at the superior or superotemporal quadrant. Mitomycin C (0.4 mg/mL) was applied on and under the scleral flap and under the Tenon’s capsule for 4 min, followed by copious irrigation with physiological saline (100 mL). Next, a deep limbal block was excised to establish a fistulous tract into the anterior chamber, and a peripheral iridectomy was performed. The scleral and conjunctival flaps were sutured with 10-0 nylon.

### 2.4. Corneal and Surgically Induced Astigmatism

Corneal astigmatism was measured preoperatively and at 6 months postoperatively using an automatic keratometer (TONOREF II; NIDEK, Aichi, Japan). Keratometric values were obtained three times at each visit, and the average of the three measurements was used for the analysis. Keratometric measurements were repeated as necessary to ensure reliable acquisition, and only values obtained under stable fixation were used for analysis. M-SIA represents the average magnitude of surgically induced astigmatism, without incorporating axis information, whereas C-SIA accounts for the magnitude and direction of astigmatism and was calculated using double-angle vector analysis. Double-angle plots displaying the individual SIA distributions were generated using the astigmatism double-angle plot tool provided on the website of the American Society of Cataract and Refractive Surgery (https://ascrs.org/tools/astigmatism-double-angle-plot-tool) (accessed on 12 December 2025) [15].

### 2.5. Data Collection

We extracted each patient’s clinical data, including age, sex, lens status, corneal astigmatism, best-corrected visual acuity (BCVA), glaucoma subtype, pre- and post-operative IOP, the number of glaucoma medications, previous surgeries, and surgery conditions. Visual acuity values were converted to the logarithm of the minimum angle of resolution (logMAR) by calculating the logarithm of the reciprocal of the corresponding decimal BCVA. Eyes with vision below the standard chart measurement were classified into four low-vision levels, with the following decimal equivalents: counting fingers, 0.00500; hand motion, 0.00250; light perception, 0.00125; and no light perception, 0.00010.

### 2.6. Outcome Measure

The primary outcome measures were comparison of SIA after BGI surgery and trabeculectomy and prognostic factors associated with SIA. These prognostic factors were evaluated using multivariate regression analyses. Secondary outcome measures included overall surgical outcomes.

### 2.7. Statistical Analysis

Univariate comparisons were performed between groups using the chi-square test, Fisher’s exact test, and Mann–Whitney U nonparametric test. We compared preoperative and postoperative data within each surgical group using the paired *t*-test. Multivariable linear regression analyses were performed using clinically prespecified (forced-entry) models to identify variables associated with M-SIA. A *p*-value < 0.05 was considered statistically significant. Statistical analysis was performed using the SPSS software (version 24.0; IBM Corp., Chicago, IL, USA).

## 3. Results

### 3.1. Patient Characteristics

This study included 109 eyes of 109 patients in the BGI group and 229 eyes of 229 patients in the trabeculectomy group. Figure S1 shows the flow of patient selection for the BGI and trabeculectomy groups. Table 1 summarizes the patients’ preoperative characteristics. All patients were Japanese. Lens status differed significantly between the two groups, with pseudophakia being more common in the BGI group. Glaucoma type also differed significantly between the two groups, with neovascular glaucoma being more common in the BGI group, whereas primary open-angle glaucoma and normal tension glaucoma were more frequently observed in the trabeculectomy group. No other significant differences were observed in patient characteristics between the two groups. Supplementary Table S1 shows the surgical conditions.

### 3.2. Outcome Measure

#### 3.2.1. Primary Outcome Measure

Table 2 shows the pre- and post-operative astigmatism outcomes. No significant difference was observed in preoperative corneal astigmatism between the two groups; however, postoperative corneal astigmatism was significantly greater in the BGI group (*p* = 0.044). Compared with the preoperative values, postoperative corneal astigmatism values worsened significantly in both groups (BGI group; *p* = 0.042, trabeculectomy group; *p* < 0.01). Moreover, the M-SIA was significantly greater in the BGI group (*p* < 0.01). Median (IQR) M-SIA was also higher in the BGI group than in the trabeculectomy group (*p* < 0.01). Because M-SIA showed a right-skewed distribution and included zero values, we performed a sensitivity analysis using log-transformed M-SIA after adding 0.01. The distribution of log(M-SIA) is shown in Figure S2.

The supplementary figures show (Figures S3–S5) the C-SIA of BGI and trabeculectomy with double-angle plots in the right and left eyes. In the BGI group (Figure S3), the double-angle plots revealed a relatively large M-SIA (1.25 ± 0.86 D and 1.61 ± 1.21 D in the right and left eyes, respectively), indicating that many eyes experienced a considerable magnitude of SIA change. However, the C-SIA remained small (0.35 D @151° ± 1.49 D and 0.27 D @169° ± 2.02 D for the right and left eyes, respectively), suggesting that the direction of SIA varied widely among individuals. BGI surgery induced relatively large, but directionally inconsistent, astigmatic changes. In the superior trabeculectomy group (Figure S4), the double-angle plots showed that the M-SIA (0.99 ± 0.86 D and 0.92 ± 0.66 D in the right and left eyes, respectively) and the C-SIA (0.16 D @ 86° ± 1.31 D and 0.25 D @ 88° ± 1.11 D for the right and left eyes, respectively) were smaller than those in the BGI group, indicating less inter-individual variability and a more stable postoperative corneal shape. The C-SIA in both eyes tended to shift toward the superior direction corresponding to the bleb location. Similarly, in the superotemporal trabeculectomy group (Figure S5), the M-SIA (1.02 ± 0.75 D and 0.80 ± 0.58 D in the right and left eyes, respectively) and the C-SIA (0.03 D @ 109° ± 1.29 D and 0.16 D @ 38° ± 0.99 D for the right and left eyes, respectively) were also smaller than those observed in the BGI group. In this subgroup, the C-SIA in both eyes tended to shift toward the superotemporal direction where the bleb was located.

Multivariable linear regression analyses were performed using prespecified forced-entry models to determine prognostic factors associated with M-SIA in all eyes (Table 3). Patients’ characteristics, including age, type of surgery, preoperative IOP, the magnitude of IOP reduction (ΔIOP, defined as the formula: preoperative IOP minus postoperative IOP), the preoperative corneal astigmatism, type of glaucoma (neovascular glaucoma/other), lens status (pseudophakia and aphakia/phakia), preoperative visual acuity, and previous vitrectomy were prespecified as possible determinants of corneal astigmatism change and simultaneously entered into the model. BGI surgery and preoperative corneal astigmatism were significantly associated with M-SIA. Undergoing BGI surgery remained a significant independent predictor of greater SIA change (Beta = 0.23, *p* < 0.01). In contrast, greater preoperative corneal astigmatism was associated with smaller SIA change (Beta = −0.19, *p* < 0.01). No other significant determinants were observed. These associations remained significant in a sensitivity analysis using log-transformed M-SIA (BGI: Beta = 0.20, *p* < 0.01; preoperative corneal astigmatism: Beta = −0.17, *p* < 0.01).

Multivariable linear regression analyses were performed using prespecified forced-entry models to determine the prognostic factors associated with M-SIA in the BGI group (Table 4). Patients’ characteristics, including age, preoperative IOP, the ΔIOP, tube insertion position (anterior chamber and sulcus/pars plana), tube patch graft (self-scleral flap/preserved sclera), preoperative visual acuity, and preoperative corneal astigmatism, were prespecified as possible determinants of corneal astigmatism change and simultaneously entered into the model. In the BGI subgroup, older age was independently associated with a greater SIA change (Beta = 0.33, *p* < 0.01), whereas greater preoperative corneal astigmatism was significantly associated with a smaller SIA change (Beta = −0.27, *p* < 0.01). Age showed the strongest influence among the included predictors. No other significant determinants were observed. These associations remained significant in a sensitivity analysis using log-transformed M-SIA (age: Beta = 0.27, *p* = 0.010; preoperative corneal astigmatism: Beta = −0.26, *p* < 0.01).

Multivariable linear regression analyses were performed using prespecified forced-entry models to determine prognostic factors associated with M-SIA in the trabeculectomy group (Table 5). Patients’ characteristics, including age, preoperative IOP, the ΔIOP, the preoperative corneal astigmatism, preoperative visual acuity, scleral flap position (superior/superotemporal), shape of the scleral flap (triangle/square), incision type of the conjunctival flap (fornix/limbus), the number of total scleral flap sutures, and the number of leftover scleral flap sutures without LSL at 6 months postoperatively were prespecified as possible determinants of corneal astigmatism change and simultaneously entered into the model. In the trabeculectomy subgroup, greater preoperative corneal astigmatism was significantly associated with a smaller SIA change (Beta = −0.15, *p* = 0.030). No other significant determinants were observed. This association was attenuated and no longer statistically significant in sensitivity analyses using log-transformed M-SIA (Beta = −0.064, *p* = 0.074).

#### 3.2.2. Secondary Outcome Measures

Table 6 presents the results for IOP, glaucoma medications, and visual acuity. In both groups, preoperative IOP decreased significantly at postoperative measurements (both groups; *p* < 0.01). Preoperative IOP was higher in the BGI group than in the trabeculectomy group, and postoperative IOP also remained higher in the BGI group. However, the ΔIOP was significantly greater in the BGI group. Glaucoma medications decreased significantly from preoperative to postoperative evaluations in both groups (both groups; *p* < 0.01). In addition, the number of preoperative medications did not differ significantly, whereas postoperative medication use was higher in the BGI group. In both groups, visual acuity (logMAR) significantly worsened post-surgery (both groups; *p* < 0.01). In the BGI group, preoperative and postoperative visual acuity were poorer.

## 4. Discussion

This study compared SIA between BGI surgery and trabeculectomy and explored prognostic factors associated with SIA. The findings demonstrated that BGI surgery induced significantly greater corneal astigmatic changes than trabeculectomy in terms of postoperative corneal astigmatism and the M-SIA. Furthermore, multivariate regression analysis identified BGI surgery as an independent factor associated with greater SIA, and eyes exhibiting lower preoperative corneal astigmatism were more likely to show larger SIA changes.

This is the first large-scale study to directly compare SIA between BGI surgery and trabeculectomy. Astigmatic changes have previously been reported after tube shunt surgery [16,17,18]; however, those studies involved small cohorts and various implant types. Therefore, by focusing exclusively on the 350-mm^2^ BGI and including a large number of eyes, our study provides a more robust and device-specific assessment of postoperative astigmatic changes.

Postoperative corneal astigmatism and M-SIA were greater in the BGI group than in the trabeculectomy group, indicating that BGI surgery is associated with greater refractive instability despite its efficacy in achieving substantial IOP reduction. Although the absolute between-group difference in postoperative corneal astigmatism was modest, the larger M-SIA after BGI surgery suggests greater corneal shape alteration. The small C-SIA in the BGI group further indicates that these changes occurred in inconsistent directions among individuals, which may reduce refractive predictability even when best-corrected visual acuity is preserved. Therefore, although the difference in postoperative astigmatism was small, the larger and more variable SIA after BGI surgery may still have clinical implications for refractive stability and subjective visual quality. Previous studies have suggested that astigmatic changes after tube shunt surgery are relatively small [16,17,18] and sometimes smaller than those observed after trabeculectomy [18]; however, this is not consistent with our findings. This discrepancy may be attributable to differences in study design, including smaller sample sizes and the use of implants with smaller endplate sizes in previous reports. In contrast, we focused exclusively on the 350-mm^2^ BGI and included a substantially larger cohort, which may have enabled more accurate detection of procedure-specific astigmatic changes. Several mechanisms may underlie why BGI surgery induces greater corneal astigmatism than trabeculectomy. First, the large endplate may exert sustained mechanical tension on the surrounding sclera and Tenon’s capsule, which may be transmitted to the anterior segment and alter corneal curvature. Second, the intraocular tube positioned near the cornea may contribute to localized biomechanical stress and asymmetric corneal deformation. Third, postoperative tissue remodeling around the endplate and tube, including fibrosis and changes in bleb morphology, may lead to progressive and directionally variable corneal shape changes. In addition, the more extensive surgical dissection required for BGI implantation compared with trabeculectomy may amplify these biomechanical effects. These factors may account for the larger and more variable SIA changes observed following BGI surgery, whereas trabeculectomy resulted in smaller and more stable astigmatic changes. The superior and superotemporal trabeculectomy subgroups demonstrated lower M-SIA values and minimal C-SIA, indicating relatively consistent postoperative corneal shape. Moreover, the direction of the C-SIA exhibited a trend of corneal steepening to the bleb location. These findings are consistent with previous reports [2,6,7,9,10,11,12,13].

Multivariate regression analyses identified baseline corneal astigmatism as a consistent and significant predictor of SIA across all surgical groups. This is consistent with a previous report on trabeculectomy [12]. Eyes with lower preoperative astigmatism exhibited larger postoperative astigmatic changes, whereas eyes with higher baseline astigmatism showed smaller induced changes, likely because corneas with more pronounced preexisting astigmatism may possess greater structural asymmetry and rigidity, rendering them less susceptible to additional surgical deformation. Conversely, corneas with relatively uniform curvature may be more vulnerable to subtle shape changes induced by surgical manipulation. Furthermore, age emerged as an independent predictor of SIA only in the BGI group, with older age being associated with larger astigmatic changes following BGI surgery. One possible explanation is that age-related stiffening of ocular tissues, including the cornea and sclera, may alter the transmission of forces generated by the large endplate and filtering bleb, as well as by the intraocular tube positioned near the cornea, leading to greater corneal deformation. Consistent with a previous report [13], age had no significant influence on SIA after trabeculectomy. This may be because the mechanical stress imposed by trabeculectomy is more localized, as the procedure does not involve a large endplate or an intraocular tube adjacent to the cornea. In addition, age and baseline corneal astigmatism remained independently associated with M-SIA in the BGI group even after log-transformed sensitivity analyses, whereas the corresponding association in the trabeculectomy group was attenuated and did not reach statistical significance (*p* = 0.074). These findings suggest that corneal astigmatic changes after BGI surgery are more robust and less sensitive to distributional assumptions than those after trabeculectomy.

This study has several limitations. First, its retrospective design may introduce selection bias. The two surgical groups differed substantially in baseline characteristics, particularly lens status, glaucoma subtype, and the history of prior vitrectomy. Although eyes undergoing intraocular surgery within six months were excluded and analyses focused on within-eye changes, prior cataract surgery and previous vitrectomy may still have influenced corneal biomechanics. To address this imbalance, glaucoma subtype, lens status, and prior vitrectomy were included in the main multivariable model; nevertheless, residual confounding cannot be fully excluded. Second, keratometric measurements were limited to anterior corneal astigmatism, and posterior corneal changes were not assessed. Although measurements were repeated to ensure reliable acquisition, automated keratometry may still be influenced by ocular surface conditions or subtle fixation instability. Evaluation of total corneal astigmatism using corneal topography would provide a more comprehensive assessment. Third, SIA was evaluated at a single postoperative time point of six months, and refractive outcomes beyond this period were not assessed. Tube opening typically occurs around the first postoperative month, and wound healing is considered relatively stable by six months; therefore, this time point was selected to evaluate pre- to postoperative astigmatic change in a clinically stable phase. Nevertheless, early postoperative changes as well as longer-term astigmatic shifts related to postoperative remodeling may not have been captured. Fourth, although prespecified multivariable models were used and sensitivity analyses with log-transformed M-SIA were performed to address right-skewed distributions, regression analyses were intended to identify associations rather than provide precise causal estimates, and the results should be interpreted with caution. Finally, minor procedural variations were present. However, fundamental surgical techniques were standardized as described in the Methods section, and case-dependent modifications such as tube position, implant type, suture management, and LSL were considered inherent to routine glaucoma surgery and within an acceptable clinical range. Prospective studies are warranted to address these limitations.

## 5. Conclusions

In conclusion, BGI surgery is associated with greater and more variable SIA than trabeculectomy. Baseline corneal astigmatism is a major determinant of postoperative astigmatic change in both procedures, while age is an additional important factor in BGI eyes. These results highlight the distinct biomechanical impact of glaucoma surgical techniques on corneal shape and underscore the importance of considering refractive outcomes when selecting surgical strategies.

## Figures and Tables

**Table 1:** Patient characteristics.

Characteristics	BGI (*n* = 109)	Trab (*n* = 229)	*p*-Value
Age, years	70.3 ± 10.6	66.7 ± 14.7	0.08
Sex, *n* (%)			0.13
Male	65 (59.6)	115 (50.2)	
Female	44 (40.4)	114 (49.8)	
Lens status, *n* (%)			<0.01
Phakia	2 (1.8)	144 (62.9)	
Pseudophakia	107 (98.2)	84 (36.7)	
Aphakia	0 (0)	1 (0.4)	
Glaucoma type, *n* (%)			<0.01
Primary open-angle glaucoma	30 (27.5)	105 (45.9)	
Exfoliation glaucoma	28 (25.7)	77 (33.6)	
Neovascular glaucoma	33 (30.3)	4 (1.7)	
Secondary glaucoma	13 (11.9)	22 (9.6)	
Primary angle closure glaucoma	5 (4.6)	6 (2.6)	
Normal tension glaucoma	0 (0)	15 (6.6)	
Previous pars plana vitrectomy, *n* (%)	39 (35.8)	2 (0.9)	<0.01

Data shown as mean ± standard deviation. BGI, Baerveldt glaucoma implant; Trab, trabeculectomy.

**Table 2:** Comparison of corneal astigmatism between the BGI and trabeculectomy groups.

Astigmatism Type	BGI (*n* = 109)	Trab (*n* = 229)	*p*-Value
Preoperative corneal astigmatism (D) ^a^	−1.30 ± 1.00	−1.04 ± 0.72	0.087
Postoperative corneal astigmatism (D) ^a^	−1.53 ± 1.01	−1.33 ± 0.92	0.044
M-SIA (D) ^a^	1.41 ± 1.05	0.94 ± 0.74	<0.01
M-SIA median (IQR) (D)	1.14 (0.75–1.76)	0.75 (0.47–1.25)	<0.01

^a^ Data shown as mean ± standard deviation. BGI, Baerveldt glaucoma implant; Trab, trabeculectomy; D, diopter; M-SIA, arithmetic mean of surgically induced astigmatism.

**Table 3:** Multivariate analysis for determining variables associated with M-SIA using a multiple regression model in all eyes.

Variable	Unstandardized Coefficient B (95% CI)	Standardized Coefficient Beta	T	*p*-Value
Type of surgery (BGI/Trab)	0.44 (0.17 to 0.70)	0.23	3.26	<0.01
Age (years)	0.002 (−0.005 to 0.010)	0.037	0.64	0.52
Preoperative IOP (mmHg)	0.004 (−0.017 to 0.025)	0.036	0.34	0.74
ΔIOP (mmHg)	0.006 (−0.014 to 0.026)	0.064	0.61	0.54
Preoperative corneal astigmatism (D)	−0.20 (−0.31 to −0.090)	−0.19	−3.58	<0.01
Type of glaucoma (neovascular glaucoma/other)	−0.070 (−0.46 to 0.32)	−0.025	−0.35	0.72
lens status (pseudophakia and aphakia/phakia)	−0.095 (−0.33 to 0.14)	−0.054	−0.79	0.43
Preoperative VA (LogMAR)	0.14 (−0.039 to 0.32)	0.085	1.54	0.13
Previous vitrectomy	−0.070 (−0.46 to 0.32)	−0.026	−0.36	0.72

BGI, Baerveldt glaucoma implant; Trab, trabeculectomy; CI, confidence interval; D, diopter; IOP, intraocular pressure; LogMAR, logarithm of minimum angle of resolution; VA, visual acuity.

**Table 4:** Multivariate analysis for determining variables associated with M-SIA using a multivariable regression model in the BGI group.

Variables	Unstandardized Coefficient B (95% CI)	Standardized Coefficient Beta	T	*p*-Value
Age (years)	0.033 (0.013 to 0.053)	0.33	3.28	<0.01
Preoperative IOP (mmHg)	0.026 (−0.026 to 0.077)	0.21	0.99	0.33
ΔIOP (mmHg)	−0.007 (−0.055 to 0.040)	−0.063	−0.30	0.77
Tube position (AC and sulcus/vitreous)	−0.057 (−0.54 to 0.42)	−0.025	−0.24	0.81
Tube patch graft (preserved/self)	−0.36 (−1.14 to 0.42)	−0.084	−0.92	0.36
Preoperative VA (LogMAR)	0.24 (−0.075 to 0.549)	0.14	1.51	0.14
Preoperative corneal astigmatism (D)	−0.28 (−0.47 to −0.094)	−0.27	−2.99	<0.01

CI, confidence interval; D, diopter; IOP, intraocular pressure; AC, anterior chamber; LogMAR, logarithm of minimum angle of resolution; VA, visual acuity.

**Table 5:** Multivariate analysis for determining variables associated with M-SIA using a multivariable regression model in trabeculectomy eyes.

Variables	Unstandardized Coefficient B (95% CI)	Standardized Coefficient Beta	T	*p*-Value
Age (years)	−0.003 (−0.010 to 0.004)	−0.068	−0.95	0.34
Preoperative IOP (mmHg)	0.004 (−0.017 to 0.026)	0.051	0.40	0.69
ΔIOP (mmHg)	0.004 (−0.016 to 0.025)	0.052	0.40	0.69
Preoperative corneal astigmatism (D)	−0.15 (−0.29 to −0.015)	−0.15	−2.19	0.030
Preoperative VA (LogMAR)	−0.008 (−0.22 to 0.20)	−0.005	−0.078	0.94
Scleral flap position (superior/superotemporal)	0.055 (−0.18 to 0.29)	0.033	0.47	0.64
Shape of the scleral flap (triangle/square)	−0.20 (−0.47 to 0.072)	−0.14	−1.45	0.15
Conjunctival flap (fornix/limbus)	0.029 (−0.22 to 0.28)	0.019	0.23	0.82
The number of scleral flap sutures	0.16 (−0.022 to 0.35)	0.16	1.73	0.084
The number of leftover scleral flap sutures without LSL	−0.007 (−0.092 to 0.078)	−0.013	−0.17	0.86

CI, confidence interval; D, diopter; IOP, intraocular pressure; LogMAR, logarithm of minimum angle of resolution; VA, visual acuity; LSL, laser suture lysis.

**Table 6:** Comparison of surgical outcomes between the BGI and trabeculectomy groups.

Surgical Outcomes	BGI (*n* = 109)	Trab (*n* = 229)	*p*-Value
Preoperative IOP (mmHg)	29.6 ± 8.5	24.6 ± 8.6	<0.01
Postoperative IOP (mmHg)	14.6 ± 4.3	13.0 ± 4.8	<0.01
ΔIOP (mmHg)	15.0 ± 9.3	11.6 ± 9.0	<0.01
Preoperative glaucoma medications	3.7 ± 1.1	3.6 ± 1.0	0.22
Postoperative glaucoma medications	1.9 ± 1.9	0.5 ± 1.2	<0.01
Preoperative VA (LogMAR)	0.64 ± 0.61	0.36 ± 0.47	<0.01
Postoperative VA (LogMAR)	0.90 ± 0.87	0.46 ± 0.55	<0.01

Data shown as mean ± standard deviation. IOP, intraocular pressure; LogMAR, logarithm of minimum angle of resolution; VA, visual acuity.

## Data Availability

The datasets generated and/or analyzed during the current study are available from the corresponding author on reasonable request.

## Supplementary Materials

The following supporting information can be downloaded at: https://www.mdpi.com/article/10.3390/jcm15041620/s1, Figure S1. Flow chart of patient selection for the Baerveldt glaucoma implant (BGI) and trabeculectomy groups. Figure S2. Histogram showing the distribution of log-transformed M-SIA in all included eyes. Figure S3: The corneal SIA of the BGI with double-angle plots in the right (*n* = 61) and left (*n* = 48) eyes; Figure S4: The corneal SIA of trabeculectomy at the superior quadrant with double-angle plots in the right (*n* = 77) and left (*n* = 92) eyes; Figure S5: The corneal SIA of trabeculectomy at the superotemporal quadrant with double-angle plots in the right (*n* = 26) and left (*n* = 34) eyes; Table S1: Surgical conditions.

## Abbreviations

The following abbreviations are used in this manuscript:

BCVA	Best-corrected visual acuity
BGI	Baerveldt glaucoma implant
IOP	Intraocular pressure
LSL	Laser suture lysis
NA	Not applicable
SIA	Surgically induced corneal astigmatism
