# Supplementary material for: Comparison of Surgically Induced Astigmatism Between Baerveldt Glaucoma Implant Surgery and Trabeculectomy: A Retrospective Cohort Study
Source: J Clin Med. 2026 Feb 20;15(4):1620. doi: 10.3390/jcm15041620 (PMC12941822; doi:10.3390/jcm15041620)
Supplement: Supplementary file 1 [file jcm-15-01620-s001.zip › jcm-4128805-supplementary.pdf]

Supplementary Information

Comparison of Surgically Induced Astigmatism Between Baerveldt Glaucoma Implant Surgery and Trabeculectomy: A Retrospective Cohort Study

Kengo Tanaka[1], Kentaro Iwasaki[1]*, Shogo Arimura[1], Marie Suzuki[1], Yoshihiro Takamura[1], Masaru Inatani[1]

[1] Department of Ophthalmology, Faculty of Medical Sciences, University of Fukui, Fukui 910-1193, Japan

***Corresponding author**

Kentaro Iwasaki

23-3 Shimoaizuki, Matsuoka, Eiheiji, Yoshida, Fukui 910-1193, Japan

Phone number: +81-776-61-8400

Fax number: +81-776-61-8131

Email address: kenkentaro0329@yahoo.co.jp

Table S1. Surgical conditions

**Conditions**	**BGI (n = 109)**	**Trab (n = 229)**
Conjunctival incision, n (%)		
Fornix based	—	76 (33.2)
Limbus based	—	153 (66.8)
Scleral flap position, n (%)		
Superior	—	169 (73.8)
Superotemporal	—	60 (26.2)
Shape of scleral flap, n (%)		
Triangle	—	110 (48.0)
Square	—	119 (52.0)
Number of scleral flaps sutures, n (%)		
5	—	5 (2.2)
4	—	13 (5.7)
3		104 (45.4)
2		107 (46.7)
Number of leftover scleral flap sutures without LSL, n (%)		
5	—	4 (1.7)
4	—	7 (3.1)
3		32 (14.0)
2		58 (25.3)
1		30 (13.1)

0		98 (42.8)
Type of implant, n (%)		
BG 101-350	83 (76.1)	—
BG 102-350	26 (23.9)	—
Tube insertion position, n (%)		
Anterior chamber	46 (42.2)	—
Sulcus	31 (28.4)	—
Pars plana	32 (29.4)	—
Tube patch graft, n (%)		
Self-scleral flap	7 (6.4)	—
Preserved sclera	102 (93.6)	—

BGI, Baerveldt glaucoma implant; Trab, trabeculectomy; LSL, laser suture lysis.

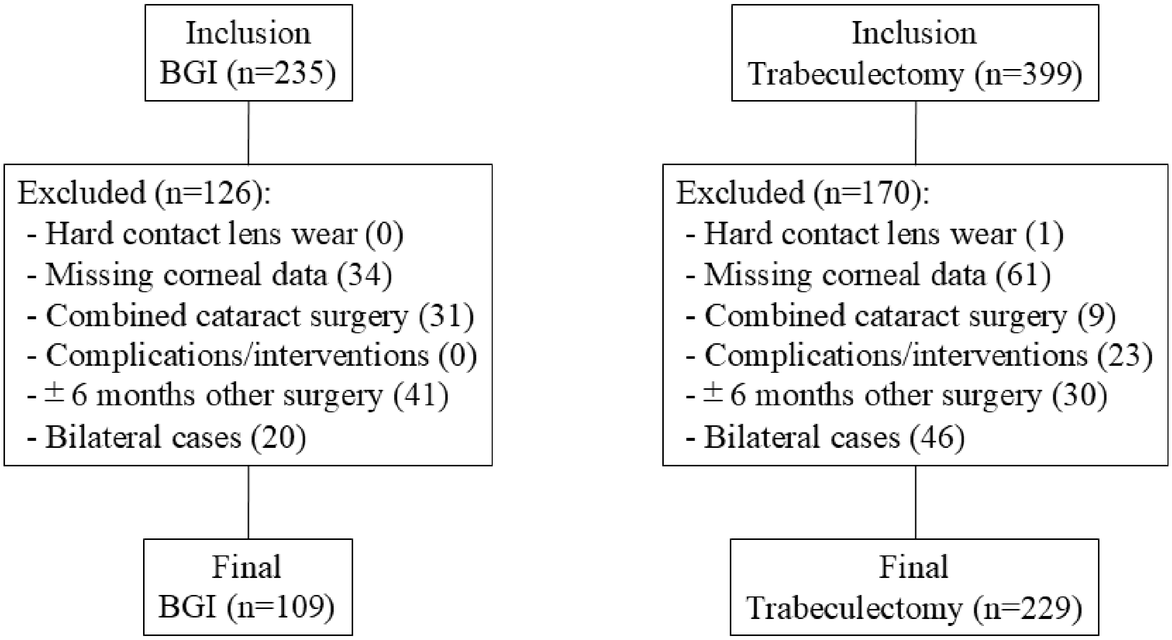

Figure S1. Flow chart of patient selection for the Baerveldt glaucoma implant (BGI) and trabeculectomy groups.

In the BGI group, 235 eyes were initially assessed for eligibility. Of these, 34 eyes were excluded because of missing corneal measurement data (lost to follow-up, n = 11; no keratometric measurement, n = 17; non-TONOREF device, n = 6), 31 eyes due to combined cataract surgery, 41 eyes because of intraocular surgery within 6 months before or after BGI surgery, and 20 eyes because of bilateral cases. Ultimately, 109 eyes were included in the final BGI analysis. In the trabeculectomy group, 399 eyes were initially assessed for eligibility. Exclusions included hard contact lens wear until immediately before surgery (n = 1), missing corneal measurement data (n = 61; lost to follow-up, n = 34; no keratometric measurement, n = 20; non-TONOREF device, n = 7), combined cataract surgery (n = 9), postoperative complications or additional procedures (n = 23; compression sutures, n = 8; additional conjunctival sutures, n = 8; epidemic keratoconjunctivitis, n = 2; corneal erosion, n = 2; additional scleral flap sutures, n = 1; fungal keratitis, n = 1; endophthalmitis, n = 1), intraocular surgery within 6 months before or after trabeculectomy (n = 30), and bilateral cases (n = 46). A total of 229 eyes were included in the final trabeculectomy analysis.

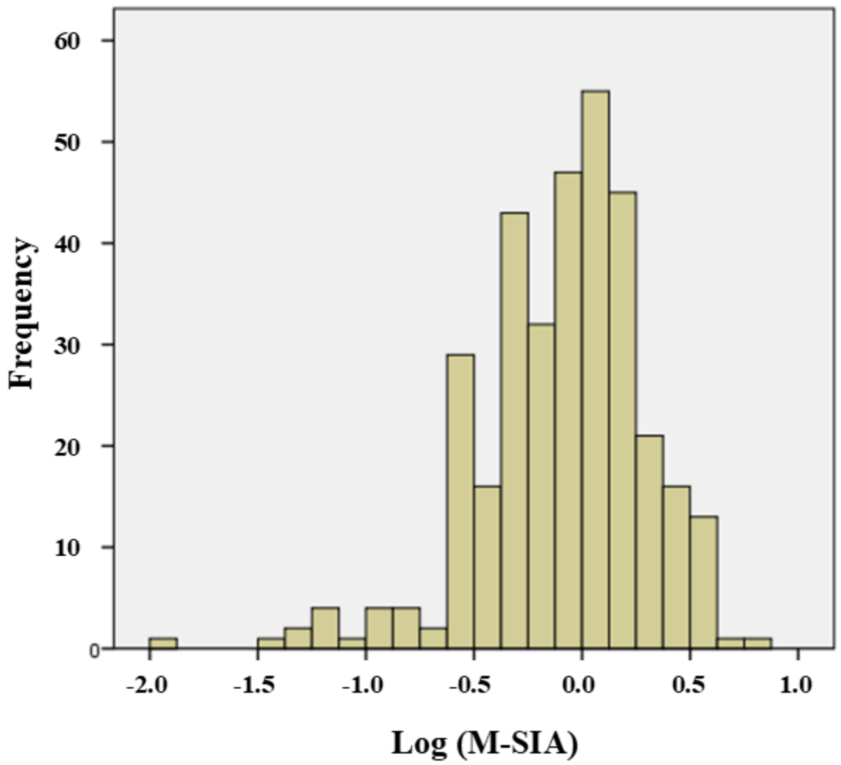

Figure S2. Histogram showing the distribution of log-transformed M-SIA in all included eyes.

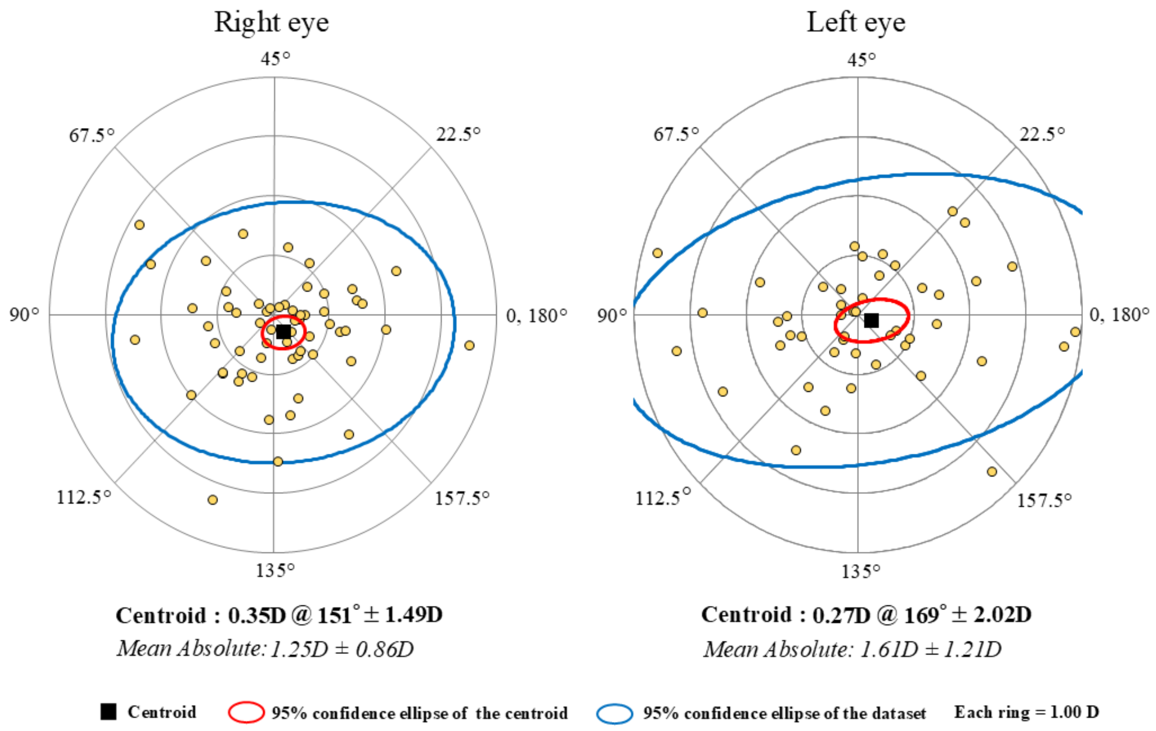

Figure S3. The corneal SIA of the BGI with double-angle plots in the right (n = 61) and left (n = 48) eyes.

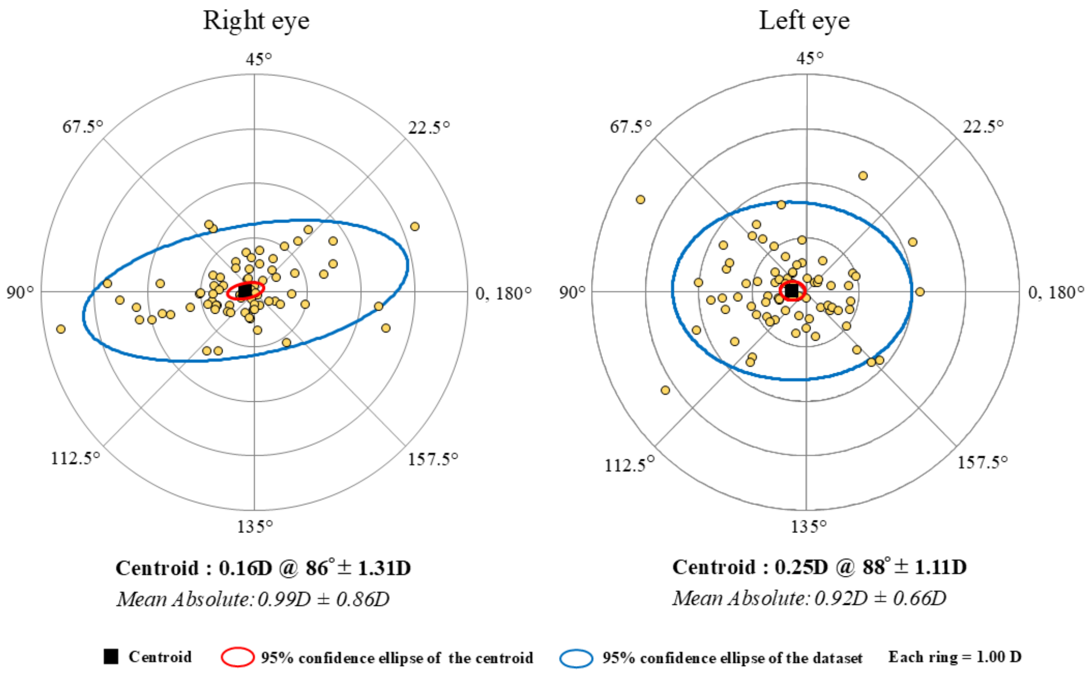

Figure S4. The corneal SIA of trabeculectomy at the superior quadrant with double-angle plots in the right (n = 77) and left (n = 92) eyes.

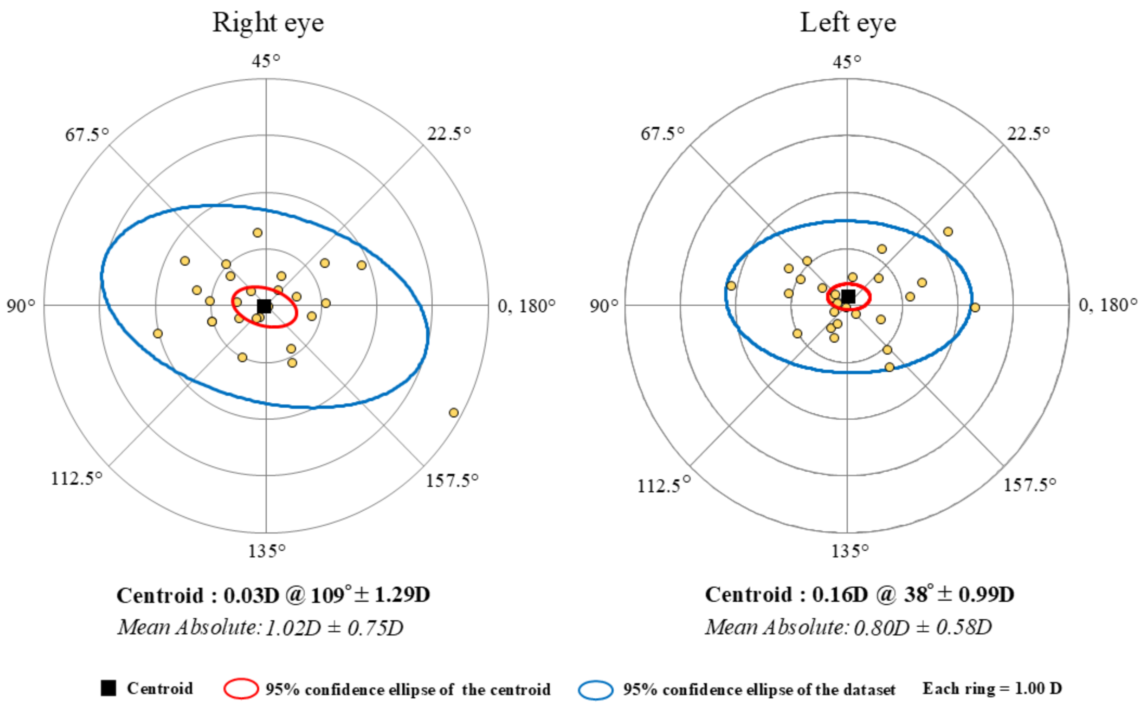

Figure S5. The corneal SIA of trabeculectomy at the superotemporal quadrant with double-angle plots in the right (n = 26) and left (n = 34) eyes.
